# A Novel Autoimmune Presentation of Wiskott‐Aldrich Syndrome: Type 1 Diabetes

**DOI:** 10.1002/iid3.70329

**Published:** 2026-02-27

**Authors:** Melanie Natasha Rayan, Yara Alshawabkeh, Samad Zia, Luma Ghalib

**Affiliations:** ^1^ Division of Endocrinology, Diabetes and Metabolism Ohio State University Medical Center Columbus Ohio USA; ^2^ Division of Internal Medicine University of Central Florida/HCA Florida North Florida Hospital Gainesville Florida USA

## Abstract

**Background:**

Wiskott‐Aldrich syndrome (WAS) is a rare X‐linked primary immunodeficiency characterized by microthrombocytopenia, eczema, and recurrent infections. While autoimmune complications are common in WAS, including autoimmune hemolytic anemia, vasculitis, and glomerulonephritis, type 1 diabetes has not been previously described.

**Case Presentation:**

We report a 37‐year‐old man with longstanding leukopenia and recurrent infections who was diagnosed with WAS after genetic testing revealed a gain‐of‐function mutation in the WAS gene. During pre‐transplant evaluation for hematopoietic stem cell transplantation, he was incidentally found to have a blood glucose level over 600 mg/dL and an A1c of 12.8%, along with classic symptoms of new‐onset diabetes. Antibody testing confirmed autoimmune diabetes with elevated GAD65 and ZnT8 antibodies, and C‐peptide of 1.2 ng/mL, with a glucose of 186 mg/dL. He was started on intravenous insulin and later discharged home on a basal‐bolus regimen.

**Conclusion:**

To our knowledge, this is the first reported case of GAD65 and ZnT8 positive autoimmune diabetes in a patient with WAS. This case expands the spectrum of autoimmune disease associated with WAS and underscores the importance of maintaining a high index of suspicion for atypical autoimmune presentations in patients with WAS.

## 1 Introduction

Wiskott‐Aldrich Syndrome (WAS) represents a rare X‐linked primary immunodeficiency with an estimated incidence of 1–9 cases per million male births [1, 2]. This hereditary disorder was first characterized in 1937 through observations of familial clustering involving three brothers with thrombocytopenia, eczematous dermatitis, bloody diarrhea, recurrent ear infections, and premature mortality [3]. The X‐linked inheritance pattern was subsequently established in 1954 [3], when a similar phenotype was observed in 16 males, but no females, over 6 generations of a single family, confirming the X‐linked nature of inheritance.

The WAS gene, located on Xp11.23, encodes the WAS protein (WASp), which is expressed in hematopoietic stem cells and plays an important role in cell signaling, migration, immune response synapse formation, phagocytosis, and autophagy, among other cellular functions [4]. Dysfunction of WASp leads to a combined immunodeficiency and microthrombocytopenia due to megakaryocyte dysfunction. The disorder is traditionally characterized by the classic triad of microthrombocytopenia, eczema, and recurrent infections, though clinical presentation varies significantly based on the degree of protein deficiency [5]. In contrast, patients with X‐linked thrombocytopenia (XLT) often have a milder phenotype dominated by thrombocytopenia and bleeding diathesis, with survival into adulthood being common. The phenotype identified in our patient, X‐linked neutropenia (XLN) due to a CRIB‐domain mutation, is considered distinct from both classical WAS and XLN. XLN usually presents early in life with severe neutropenia, recurrent bacterial infections, and, in some cases, marrow dysplasia or transformation. Although survival may extend into adulthood, affected individuals are at risk for significant infectious complications, autoimmunity, and hematologic malignancy in the absence of curative therapy [6]. According to comprehensive genotype–proteotype studies, missense mutations in exons 7–11, including exon 9, typically result in reduced but detectable WASp expression in lymphocytes [7, 8, 9]. Quantitative protein studies demonstrate that WAS levels in patients with such missense mutations are decreased compared to normal controls, but not absent.

The clinical manifestations of WAS exist along a broad spectrum, from isolated thrombocytopenia to severe immunodeficiency with autoimmunity and hematologic malignancy [5]. Autoimmune complications in WAS are heterogeneous and affect multiple organ systems, with well‐recognized manifestations including autoimmune hemolytic anemia, vasculitis, inflammatory arthritis, IgA nephropathy, and inflammatory bowel disease. These complications are often seen in more severe disease phenotypes and can significantly contribute to overall morbidity. Here, we present a rare case of type 1 diabetes (T1D) in a patient with WAS, highlighting the complex evolving spectrum of immune dysregulation associated with this disorder.

### 1.1 Case Presentation

We present a 37‐year‐old male with a longstanding history of chronic infections including upper respiratory, ear infections, sinus infections, pneumonia, and cytopenias dating back to childhood. There is no known family history of type 1 diabetes or autoimmune endocrinopathies. Relevant family history includes autoimmune disease in a paternal uncle (multiple sclerosis), hematologic and bleeding disorders in paternal grandparents, and malignancies including kidney cancer in the father, brain tumor in a brother, esophageal cancer in the maternal grandfather, breast cancer in a maternal aunt, and melanoma in a paternal uncle. At age 17, evaluation for persistent leukopenia and natural killer cell dysfunction included a bone marrow biopsy which revealed neutrophil maturation arrest in the band stage and multiple large granular lymphocytes, though no definitive diagnosis was established at that time. His medical history also included hypertension, vitamin D deficiency, iron deficiency anemia, and type III paraesophageal hernia with Cameron ulcers.

Ten years later, given his history of recurrent infections and immunodeficiency, workup for chronic viral infections including HIV and hepatitis was performed and was negative. Imaging revealed splenomegaly measuring 14.9 cm. Following a 6‐year lapse in care due to insurance issues, he returned with progressive symptoms including rectal bleeding and severe iron deficiency with ferritin of 3.9 ng/mL and transferrin saturation of 2%. During this period, he developed worsening transaminitis and underwent extensive evaluation including testing for w1ilson's disease, hereditary hemochromatosis, and autoimmune hepatitis, all of which were negative. No systematic screening for other autoimmune diseases was performed at this time. A liver biopsy showed only mild steatosis and minimal portal inflammation without fibrosis. The patient was not receiving intravenous immunoglobulin (IVIG) replacement therapy and was not on antimicrobial prophylaxis at the time of presentation or during follow‐up.

The platelet count was markedly reduced at 92 × 10³/µL (reference 142–508 × 10³/µL). The absolute neutrophil count was also reduced at 0.9 × 10³/mm³ (reference 1.6–6.2 × 10³/mm³). Immunoglobulin levels demonstrated low IgA (< 10 mg/dL, reference 61–356 mg/dL), markedly reduced IgE (<2 IU/mL, reference 0–103 IU/mL), normal IgG (1223 mg/dL, reference 767–1590 mg/dL), and normal IgM (116 mg/dL, reference 37–286 mg/dL). The lymphocyte percentage was 43.1% (reference 9.0%–51.0%). Flow cytometry (FISH) identified atypical, immature myeloblasts (CD34+, CD13+, CD33+, CD117+) comprising 3.7% of the total cell population. In addition, small subpopulations of T lymphocytes were observed, including CD3+/CD4−/CD8− (3.4%) double‐negative T cells (a finding sometimes associated with autoimmune disorders) and CD3+/CD4+/CD8+ (1.6%) double‐positive T cells. B cells included a CD19+/CD10+ subset lacking kappa/lambda expression. Given his persistent cytopenias and clinical deterioration, a bone marrow biopsy was performed demonstrating profound hypocellularity of 5%–10% with decreased but intact trilineage hematopoiesis and no evidence of clonal disease or dysplasia. Genetic testing revealed a mutation in exon 9 of the WAS gene, within the CRIB (Cdc42/Rac binding) domain of the WAS protein. WAS, Exon 9, c.869T>C (p.Ile290Thr) hemizygous was identified. T‐cell subset analysis showed low CD4⁺ T helper cells at 8.4% (reference 32%–62%) and absolute count 31/mm³ (reference 266–2213/mm³). CD8⁺ T suppressor cells were 13.4% ([10, 12] 44%) with absolute count 50/mm³ (reference 91–1428/mm³). The CD4/CD8 ratio was 0.6. Given the risk of malignant transformation and progressive cytopenias, he was deemed a candidate for allogeneic hematopoietic stem cell transplantation (HSCT). High‐resolution HLA typing performed as part of pre‐transplant evaluation revealed the following alleles: HLA‐A 02:01/26:01, HLA‐B 07:02/27:05, HLA‐C 01:02/07:02, HLA‐DRB1 01:01/07:01, HLA‐DQB1 02:02/05:01, HLA‐DQA1 01:01/02:01, HLA‐DPB1 02:01/04:01.

During pre‐transplant evaluation at the hematology transplant clinic, the patient was incidentally found to have a random blood glucose of 488 mg/dL (RR, < 140 mg/dL), with a repeat point‐of‐care glucose of 572 mg/dL. He subsequently presented to the emergency department where his glucose was greater than 600 mg/dL. He was not receiving corticosteroids at the time of presentation. The patient reported a several‐week history of polydipsia, polyuria, and profound fatigue requiring 4–5 h daily naps. He had experienced significant unintentional weight loss of approximately 20 pounds over the preceding 2 months, but denied abdominal pain, nausea or vomiting. His family history was notable for type 2 diabetes mellitus in his mother diagnosed at age 40.

Laboratory evaluation revealed a hemoglobin A1c of 12.8% (RR, < 5.7%), representing a dramatic increase from 6.5% obtained 8 months prior. Additional studies showed beta‐hydroxybutyrate of 1.81 mmol/L (RR, < 0.6 mmol/L), urine ketones of 15 mg/dL, urine glucose greater than 1000 mg/dL (RR, 0–15 mg/dL), sodium of 124 mEq/L (RR, 135–145 mEq/L), bicarbonate of 27 mEq/L (RR, 22–26), and anion gap of 19 mEq/L (RR, 2–12 mEq/L) without significant acidosis. His white blood cell count was 2.51 × 10³/μL (RR, 4.0–11.0 × 10³/μL) and platelet count was 131 × 10³/μL (RR, 150–400 × 10³/μL). Given the unusual presentation of new‐onset diabetes, comprehensive autoantibody testing was performed which revealed positive GAD65 antibodies at 3.90 U/mL (RR, < 5.0 U/mL) and markedly elevated ZnT8 antibodies greater than 500 U/mL (RR, < 15 U/mL). IA‐2 antibodies were normal at 0.02 U/mL (RR, < 7.5 U/mL), insulin autoantibodies were negative at 0.00 U/mL (RR, < 0.4 U/mL), and anti‐pancreatic islet cell antibodies were negative. C‐peptide level was 1.23 ng/mL (RR, 0.5–2.0 ng/mL) with a concurrent glucose of 186 mg/dL.

The patient was initially managed with continuous intravenous insulin and within 24 h was successfully transitioned to a basal‐bolus insulin regimen consisting of 12 units of insulin glargine daily with mealtime coverage with 4 units of Lispro with large meals and 2 units of Lispro with small meals. He was discharged with endocrinology follow‐up for ongoing diabetes management. Post‐transplant, the patient is on basal‐bolus insulin therapy with insulin glargine 8 units every morning and correction with insulin lispro (1 unit per 50 mg/dL above 200 mg/dL before meals as needed). Post‐transplant blood glucose generally ranged between 88 and 191 mg/dL, representing a substantial improvement compared with the pre‐transplant HbA1c of 12.8%, which reflected poor glycemic control. Subsequently, he underwent matched unrelated donor allogeneic HSCT using fludarabine, busulfan, and antithymocyte globulin conditioning with graft‐versus‐host disease prophylaxis using tacrolimus and methotrexate. During the transplant admission, he received high‐dose methylprednisolone at 80 mg intravenously before and during antithymocyte globulin infusions, requiring careful diabetes management in the context of worsening hyperglycemia due to corticosteroids. He is showing steady hematologic improvement and remains under close surveillance for immune system reconstitution and sustained diabetes care. Hematologic parameters before and after transplant demonstrated early HSCT effects. Pre‐transplant: WBC count 2.1 × 10³/µL (reference 3.73–10.10 × 10³/µL), platelet count 92 × 10³/µL (reference 146–337 × 10³/µL), absolute neutrophil count 0.9 × 10³/µL (reference 1.57–6.19 × 10³/µL), and absolute lymphocyte count 2.0 × 10³/µL (reference 0.83–3.57 × 10³/µL). Post‐transplant: WBC count 6.02 × 10³/µL, platelet count 81 × 10³/µL, absolute neutrophil count 5.37 × 10³/µL, and absolute lymphocyte count 0.22 × 10³/µL. These findings demonstrate successful neutrophil recovery and normalization of total leukocyte count, while persistent thrombocytopenia and lymphopenia reflect the expected pattern of gradual immune reconstitution following HSCT.

## 2 Discussion

Our case represents the first documented association between WAS and T1D, revealing an unexplored feature of autoimmune dysregulation in this complex immunodeficiency disorder. Notably, the patient's HLA typing did not reveal high‐risk class II alleles commonly associated with type 1 diabetes (such as DR3‐DQ2 or DR4‐DQ8 haplotypes), indicating no known HLA‐based genetic predisposition to T1D. This novel finding broadens the recognized spectrum of autoimmunity in WAS and raises important implications for clinical monitoring and management. The patient′s clinical and laboratory evaluation considered prolonged or repeated infections and medications as possible contributors to immune dysregulation and autoimmune complications. At the time of evaluation there was no evidence of ongoing or chronic infection; infectious work‐up, including blood cultures and viral serologies, was unremarkable. The patient was not on medications known to affect immune function (e.g., immunosuppressants, corticosteroids, or biologics) prior to transplant. Autoimmune manifestations, including autoimmune hepatitis and dysregulated glycemic control, were observed in the absence of these confounding factors, supporting the attribution to the underlying WAS mutation rather than external causes.

Autoimmune manifestations have long been recognized as a hallmark of severe WAS, affecting a substantial proportion of patients and contributing significantly to morbidity and mortality [11]. The immune dysregulation characteristic of WAS is marked by impaired regulatory T cell activity, heightened B cell responsiveness, and persistent inflammatory signaling, all of which contribute to the development of autoimmune complications. Commonly reported manifestations include autoimmune hemolytic anemia, vasculitis, arthritis, immune‐mediated neutropenia, and renal involvement such as glomerulonephritis and IgA nephropathy.

To facilitate standardized clinical characterization of affected individuals, a 5‐point severity scoring system has been established, with higher scores reflecting more severe disease [10, 11]. A score of 0 corresponds to individuals with X‐linked neutropenia or myelodysplasia who lack other clinical manifestations. Scores of 1 and 2 are indicative of X‐linked thrombocytopenia (XLT), a milder form of the disease characterized by isolated thrombocytopenia (score 1) or thrombocytopenia accompanied by mild or transient eczema and infrequent infections (score 2). Patients assigned a score of 3 typically present with thrombocytopenia along with persistent eczema and recurrent infections that respond to treatment. A score of 4 denotes more severe disease with refractory eczema or infections. The most severe phenotype, represented by a score of 5, is defined by the presence of autoimmunity and/or malignancy in addition to thrombocytopenia and other core features [12]. Autoimmunity, in particular, is a hallmark of this advanced disease stage and may present with a wide range of immune‐mediated manifestations.

Despite this well‐characterized autoimmune propensity, pancreatic autoimmunity has remained absent from the WAS literature. Large clinical series and comprehensive reviews examining autoimmune complications in WAS have not included T1D, either as a common feature or as an infrequent association [4, 11, 13, 14, 15]. Major consensus guidelines, such as the Practice Parameter for the Diagnosis and Management of Primary Immunodeficiency published by the American Academy of Allergy, Asthma, and Immunology, also do not reference T1D in their discussion of autoimmune complications in WAS [16]. This absence is particularly notable given the wide range of organ‐specific autoimmune conditions involving the hematologic, renal, and gastrointestinal systems that are well described in this disorder.

Other monogenic immunodeficiencies marked by regulatory T‐cell (Treg) dysfunction most notably Immune dysregulation, Polyendocrinopathy, Enteropathy, X‐linked syndrome (IPEX) and Autoimmune Polyendocrinopathy‐Candidiasis‐Ectodermal Dystrophy (APECED) frequently present with T1D. IPEX, caused by Forkhead box P3 (FOXP3) defects, typically manifests early in life with a triad of enteropathy, eczema, and insulin‐dependent T1D [17]. APECED, resulting from central tolerance failure due to Autoimmune Regulator (AIRE) mutations, includes endocrine autoimmunity, with T1D observed in 1%–18% of cases across different cohorts [18]. These syndromes underscore how monogenic defects in immune regulation can specifically target pancreatic beta cells. While WAS also involves Treg abnormalities, T1D has not been previously reported, emphasizing the novelty of our case and expanding the recognized autoimmune phenotype of WAS.

Documenting this rare occurrence offers new insight into the broader spectrum of immune dysregulation in WAS, suggesting that autoimmunity in these patients may extend beyond traditionally recognized manifestations. The presence of autoimmune disease not only reflects more severe immune dysfunction but also often accelerates the indication for HSCT and necessitates careful peri‐transplant evaluation to optimize outcomes [16]. In our case, routine pre‐transplant evaluation uncovered previously unrecognized T1D, underscoring the value of vigilance in recognizing symptoms of T1D in patients with WAS.

This finding suggests that systematic investigation of diabetes prevalence in larger WAS cohorts may be warranted. HSCT is the definitive treatment for WAS, aimed at correcting the underlying immunodeficiency and associated immune dysregulation. Notably, HSCT has also been studied in individuals with newly diagnosed T1D without primary immunodeficiency. Multiple studies of autologous HSCT in new‐onset T1D have shown that a substantial proportion of patients can achieve prolonged insulin independence and improved β‐cell function, with remission lasting months to years, particularly when performed soon after diagnosis while residual beta cell function is present [19, 20, 21, 22]. These findings suggest that HSCT may transiently preserve or partially restore beta‐cell function in the context of autoimmune diabetes. However, it is important to note that HSCT is not standard of care for T1D outside of research settings. Although such effects have not been evaluated in patients with WAS, our case featuring the incidental diagnosis of ZnT8‐ and GAD65‐positive T1D during pre‐HSCT evaluation for WAS raises the question of whether immune reconstitution in this setting might similarly influence autoimmune diabetes. This case highlights a potential overlap between therapeutic immune reset for immunodeficiency and modulation of concurrent autoimmune disease. As our understanding of WAS continues to evolve, this case underscores the importance of maintaining clinical awareness for novel autoimmune manifestations that could impact therapeutic approaches and long‐term outcomes in this complex immunodeficiency syndrome.

## 3 Conclusion

This case highlights autoimmune diabetes as a previously unrecognized feature of WAS, expanding its known autoimmune spectrum and underscoring the importance of considering atypical autoimmune manifestations in affected patients, findings that may modify future screening and management approaches.

## Author Contributions

Melanie Natasha Rayan contributed to data acquisition, literature review, and manuscript writing. Yara Alshawabkeh contributed to literature review and manuscript writing. Samad Zia contributed to data interpretation and manuscript revision. Luma Ghalib provided supervision of the project and guided manuscript revision.

## Ethics Statement

Written informed consent was obtained from the patient for publication of this case report.
